# Shoulder Musculoskeletal Disorder Rehabilitation Using a Robotic Device Based on Electromyography (EMG) Biofeedback: A Retrospective Cohort Study

**DOI:** 10.3390/medicina61020272

**Published:** 2025-02-05

**Authors:** Martin Lavallière, Mathieu Tremblay, Etienne Ojardias, Maxime Turpin, Anaïck Perrochon, Philippe Rigoard, Lisa Goudman, Maarten Moens, Romain David, Maxime Billot

**Affiliations:** 1 Program of Kinesiology, Department of Health Sciences, Université du Québec à Chicoutimi (UQAC), Saguenay, QC G7H 2B1, Canada; 2 Laboratoire de Recherche Biomécanique & Neurophysiologique en Réadaptation Neuro-Musculo-Squelettique—Lab BioNR, Université du Québec à Chicoutimi (UQAC), Saguenay, QC G7H 2B1, Canada; 3 Program of Kinesiology, Department of Health Sciences, Université du Québec à Rimouski (UQAR), Rimouski, QC G5L 3A1, Canada; 4 Physical Medicine and Rehabilitation Department, University Hospital of Saint-Etienne, 42100 Saint-Etienne, France; 5 Lyon Neuroscience Research Center, Trajectoires Team (Inserm UMR-S 1028, CNRS UMR 5292, Lyon1 & Saint-Etienne Universities), 42270 Saint-Etienne, France; 6 ILFOMER (Institut Limousin de Formation aux Métiers de la Réadaptation), Université de Limoges, 87000 Limoges, France; maximeturpin36@hotmail.fr (M.T.);; 7 HAVAE UR20217 (Handicap, Ageing, Autonomy, Environment), University of Limoges, 87000 Limoges, France; 8 CHU de Poitiers, PRISMATICS Lab (Predictive Research in Spine/Neuromodulation Management and Thoracic Innovation/Cardiac Surgery), 86000 Poitiers, France; 9 CHU de Poitiers, Department of Neurospine Surgery & Neuromodulation, 86000 Poitiers, France; 10 Prime Institute UPR 3346, CNRS, ISAE-ENSMA, Université de Poitiers, 86360 Chasseneuil-du-Poitou, France; 11 Department of Neurosurgery, Universitair Ziekenhuis Brussel, 1090 Jette, Belgium; 12 STIMULUS Consortium (Research and Teaching Neuromodulation VUB/UZ Brussel), Vrije Universiteit Brussel, 1090 Brussels, Belgium; 13 Center for Neurosciences (C4N), Vrije Universiteit Brussel, 1090 Brussels, Belgium; 14 Pain in Motion (PAIN) Research Group, Department of Physiotherapy, Human Physiology, and Anatomy, Faculty of Physical Education and Physiotherapy, Vrije Universiteit Brussel, 1090 Brussels, Belgium; 15 Research Foundation—Flanders (FWO), 1090 Brussels, Belgium; 16 Department of Radiology, Universitair Ziekenhuis Brussel, Laarbeeklaan 101, 1090 Brussels, Belgium; 17 Physical and Rehabilitation Medicine Unit, Poitiers University Hospital, University of Poitiers, 86021 Poitiers, France; 18 Centre de Recherche sur la Cognition et l’Apprentissage UMR7295, CNRS, Université de Poitiers, Université François Rabelais de Tours, 86000 Poitiers, France

**Keywords:** rehabilitation, shoulder, electromyography feedback, visual biofeedback, assistive robot, musculoskeletal disorder

## Abstract

*Background and Objectives*: While shoulder injuries represent the musculoskeletal disorders (MSDs) most encountered in physical therapy, there is no consensus on their management. In attempts to provide standardized and personalized treatment, a robotic-assisted device combined with EMG biofeedback specifically dedicated to shoulder MSDs was developed. This study aimed to determine the efficacy of an 8-week rehabilitation program (3 sessions a week) using a robotic-assisted device combined with EMG biofeedback (RA-EMG group) in comparison with a conventional program (CONV group) in patients presenting with shoulder MSDs. *Materials and Methods*: This study is a retrospective cohort study including data from 2010 to 2013 on patients initially involved in a physical rehabilitation program in a private clinic in Chicoutimi (Canada) for shoulder MSDs. Shoulder flexion strength and range of motion were collected before and after the rehabilitation program. Forty-four patients participated in a conventional program using dumbbells (CONV group), while 73 completed a program on a robot-assisted device with EMG and visual biofeedback (RA-EMG group); both programs consisted of two sets of 20 repetitions at 60% of maximal capacity. *Results*: We showed that the RA-EMG had significantly greater benefits than the CONV group for shoulder flexion strength (4.45 [2.6;6.15] kg vs. 2.3 [0.90;4.775] kg, U = 761, *p* = 0.013) and for normalized strength (77.5 [51.3;119.1] % vs. 39.1 [16.6;89.2] %, U = 755, *p* = 0.016). In addition, the RA-EMG group showed a trend to greater absolute gain of ROM than the CONV group (10.0 [0;24.3] degrees vs. 5.5 [0;12.0] degrees, U = 1931, *p* = 0.067), and a greater benefit in normalized ROM was observed for the RA-EMG (7.4. [0;17.7] %) than the CONV group (4.6 [0;10.8], U = 1907, *p* = 0.046). *Conclusions*: The current retrospective cohort study showed that a specific and tailored 8-week rehabilitation program with constant effort by automatic adjustment of the level of resistance by EMG feedback induced greater benefits for shoulder flexion strength and a trend to improve range of motion compared to conventional rehabilitation in patients with shoulder MSDs. Future research should be pursued to determine the added potential of this approach for abduction and external rotation with a randomized controlled design.

## 1. Introduction

Shoulder injuries, such as tendinopathies, subacromial pain syndrome and rotator cuff-related shoulder pain [1], represent the most widely encountered musculoskeletal disorders (MSDs) in physical therapy [2]. Physical work condition has been identified as one of the major causes of MSDs [3,4], especially at the shoulder [2,4]. While shoulder MSDs result in functional discomfort associated with pain [4,5,6] and impact quality of life and work productivity [7], management ranging from a surgical approach [7,8,9,10] to conservative treatment such as rest periods, analgesics, pharmacological therapy and physical therapy are still debated [11,12].

To date, surgery does not appear to be more effective than physical therapy for subacromial pain syndrome [7,8,9,12], and of course, it is more invasive and expensive. A recent systematic review and meta-analysis reported that five treatments (acupuncture, exercise, exercise plus manual therapy, laser therapy and Transcutaneous Electrical Nerve Stimulation (TENS)) had a high effect size (surface under the cumulative ranking curve values >50%) for management of pain and functional outcomes in subacromial shoulder conditions at short-term follow-up (2–6 weeks) [13]. In addition, exercise therapy has been shown to improve the active range of motion, overall shoulder function and pain scores at short- and long-term follow-up in patients presenting with subacromial pain syndrome [5,13,14,15,16,17,18]. Despite these promising benefits, there is no strong evidence to delineate the contour of dose–response efficacy, including number of repetitions, frequency and level of effort [19]. Attempting to help clinicians provide a standardized and safe approach, muscle strengthening machines have undergone major technological changes, leading to the appearance of a generation of machines integrating computerization, automation and robotic assistance.

In this context, robot-assisted training has been developed in neuro-rehabilitation [20,21,22,23,24,25,26,27]. A meta-analysis by Chen et al. [20] showed that robot-assisted training provided better outcomes for motor impairment disability than therapist-assisted training and no inferior outcomes for upper limb capacity, activity of daily living and social participation after stroke. Other studies using robotic-assisted devices for the rehabilitation of humerus [28,29] or radius fractures [30] have shown promising results. To reinforce the benefits of a robotic-assisted program for the upper limbs [31], electromyography (EMG) activity biofeedback has been combined to help patients reach a target through visual feedback and to adjust the level of assistance [32]. Using a robotic-assisted device combined with EMG biofeedback in an 8-week rehabilitation program (3 sessions a week), Bui et al. [33] showed significant improvement in maximal voluntary isometric flexion and abduction contraction of the left and right shoulders in a healthy population (n = 7). A robotic-assisted program combined with the EMG biofeedback approach has yet to be evaluated in the rehabilitation of patients presenting with shoulder MSDs.

The objective of this study was to determine the efficacy of an 8-week rehabilitation program using a robotic-assisted device combined with EMG biofeedback (RA-EMG group) compared to a conventional program (CONV group) in patients presenting with shoulder MSDs after occupational injury. We hypothesized that a robotic-assisted device combined with EMG biofeedback would add value to conventional training programs in patients with MSDs.

## 2. Materials and Methods

### 2.1. Participants

**Figure 1:**
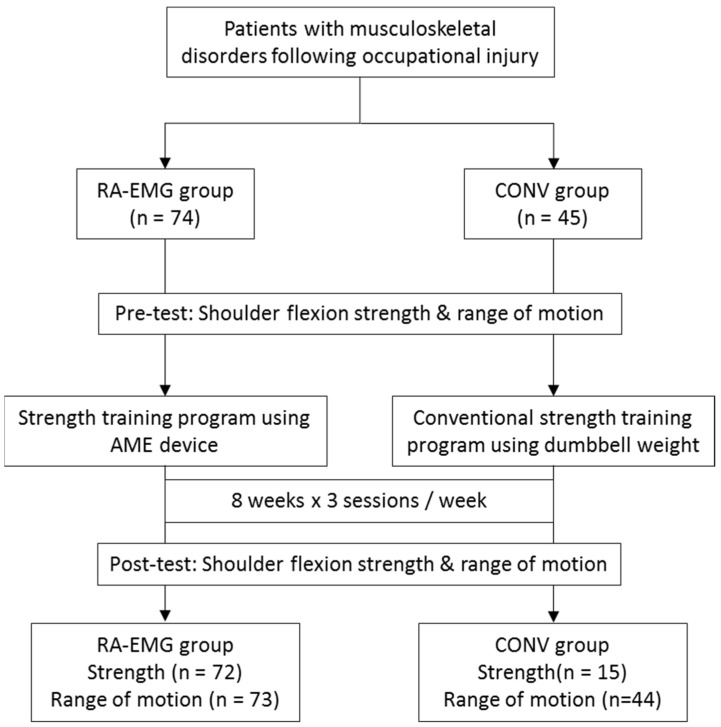
Study design and flow chart. RA-EMG group: robotic-assisted with electromyography feedback (AME: Appareil Multifonctionnel d’Entraînement—multifunctional training device); CONV group: conventional training.

This study is a retrospective cohort study including data from 2010 to 2013. Patients were initially involved in a physical rehabilitation program in a private clinic in Chicoutimi (Canada). To be included in this study, the participants had to be diagnosed with shoulder MSDs (i.e., subacromial pain syndrome, shoulder dislocation, adhesive capsulitis, etc.) following an occupational injury; be referred by a health professional (medical doctor, orthopedic physician or physiotherapist); able to practice physical activities without medical contraindications; have completed 3 training sessions a week during 8 weeks; and have completed the training with the robotic-assisted device combined with EMG-FB (RA-EMG group) or the conventional training program (CONV group) (Figure 1). The exclusion criteria were patients with behavioral (cognitive and/or psychiatric) disability. Patients were allocated to RA-EMG or CONV groups based on clinical practice, without randomization. All of the subjects gave their informed consent to participate in the study. The study was conducted in accordance with the Declaration of Helsinki and was approved by the Ethics Committee of Research of the Université du Québec à Chicoutimi (602-545-01).

### 2.2. Experimental Protocol

**Figure 2:**
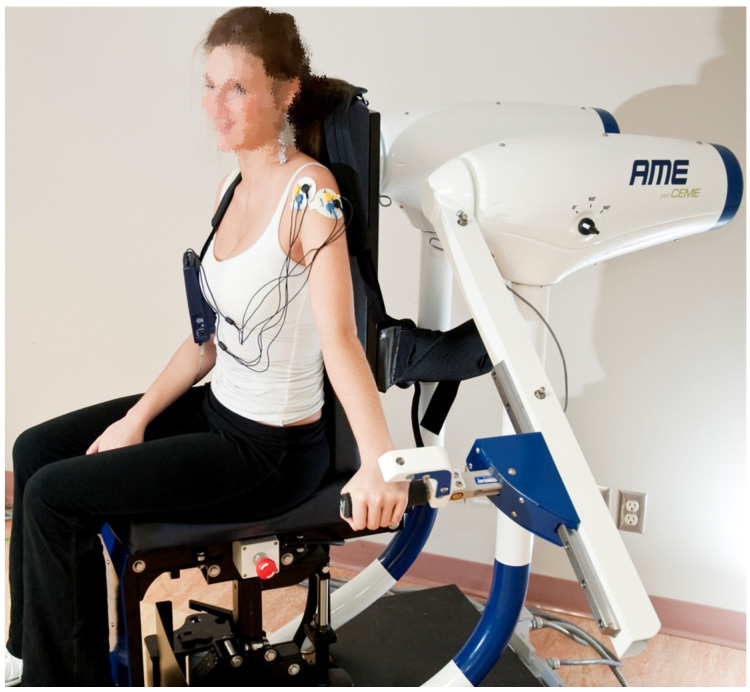
The AME device (Appareil Multifonctionnel d’Entraînement—multifunctional training device): a patient is conducting an abduction exercise with the placement of surface EMG electrodes on the shoulder.

Before and after the training program, all patients performed pre- and post-test measurements of shoulder flexion strength with a voluntary maximal isometric contraction with the shoulder positioned at 5° of flexion (0° corresponding to reference anatomical position) and with the elbow in full extension. Strength measurements were carried out with the robotic-assisted device AME (Appareil Multifonctionnel d’Entraînement—multifunctional training device) (CEME, Saguenay, QC, Canada) (American Certificate US.8.262.541, US.8.187.152; Canadian certificate CA 2714914) (Figure 2) for the RA-EMG group and with a dynamometer for the CONV group. Active shoulder flexion range of motion was collected using a manual goniometer. Thereafter, the patients completed a training program, with the conventional approach or with the robotic-assisted device combined with EMG biofeedback, during 8 consecutive weeks of 3 sessions a week. Each training session started with a 10 min warm-up period of aerobic exercise (treadmill, cycle ergometer or stair climber machine) at low to moderate intensity (rated 1 to 4 on the modified Borg scale) to attain a steady state [34]. To complete the warm-up, a 5 min exercise consisting of voluntary shoulder movement was adjusted to individual functional limitations in the frontal, sagittal and transversal planes without any external intervention. After this, each group performed specific training with the AME or conventional approach.

### 2.3. Training Programs

#### 2.3.1. RA-EMG Group

The RA-EMG group carried out an 8-week rehabilitation reinforcement training program using the AME device with 2 sets of 20 repetitions for 3 sessions a week. A 1 min 30 s rest period was observed between sets [35].

The patient was seated, and the axis of shoulder rotation aligned with the axis of rotation of the AME device. The position parameters were stored in the device interface, which reproduced settings between sessions [36]. Surface EMG electrodes (Thought Technology Ltd., Montréal, QC, Canada) were placed on the anterior deltoid [37,38]. A two-channel Myotrac Infinity Encoder (Thought Technology Ltd., Montréal, QC, Canada) monitored the EMG activity during training sessions. Data acquisition was performed at a frequency of 2048 Hz and collected by the internal computer of the AME device.

Maximal isometric shoulder flexion was performed before starting each training session to determine the maximal EMG activity. The exercises included performing shoulder flexion and extension from 0–30° to a maximum of 90°. Movements were performed at a level of 60% of the maximal EMG activity displayed on a screen in front of the patient. The speed control system of the AME device adjusted the load based on the EMG activity.

#### 2.3.2. CONV Group

The CONV group performed an 8-week strength training program using dumbbell weights consisting of 2 sets of 20 repetitions with a rest period of 1 min 30 s between sets, for 3 sessions a week. Dumbbell weight loads were adjusted based on patient capacity throughout the program based on 60% of the maximal capacity. The two shoulders were randomly trained. The exercises were elbow flexion, shoulder anteflexion, shoulder abduction and shoulder elevation.

### 2.4. Outcomes

#### 2.4.1. Absolute and Normalized Shoulder Flexion Strength Gain

Absolute shoulder flexion strength gain was assessed with a voluntary maximal isometric contraction of shoulder flexion. Absolute strength gain was calculated between the pre- and post-session (Absolute Strength gain (kg) = Post strength − Pre-test strength).

The normalized shoulder flexion strength gain is a percentage from the pre-test session. (Normalized strength gain (%) = (Absolute strength gain/Pre-test strength × 100)). This variable compensates for an amplitude of strength produced by each person and refers to a percentage increase of strength produced at the end of the program.

#### 2.4.2. Absolute and Normalized Shoulder Flexion Range of Motion Gain (ROM)

The absolute shoulder flexion ROM gain of each patient was calculated with the help of a manual goniometer. The amplitude gain was estimated between the program’s first and last session: ROM gain = Post-test ROM − Pre-test ROM.

To calculate the normalized shoulder flexion ROM gain, we divided the absolute amplitude by the maximum absolute amplitude at the pretest and multiplied by 100 (Normalized ROM gain = (Absolute ROM gain/Pre-test ROM × 100)).

### 2.5. Statistical Analysis

Groups are presented using mean values and standard deviations for quantitative variables and by numbers and percentages for qualitative variables. The t test for independent measure was performed for demographic characteristics. The statistical analysis assessed the effects of two 8-week strength rehabilitation programs (CONV versus RA-EMG) on strength gain and ROM in patients presenting with shoulder MSDs (SigmaPlot, version 12.5, Systat Software Inc., Palo Alto, CA, USA). The Shapiro–Wilk test tested normality of the data. The Mann–Whitney test was performed for strength and amplitude outcomes. Data were expressed as the median and the Interquartile Range (IQR). The t test for independent measure was performed for demographic characteristics. *p* < 0.05 was considered statistically significant.

## 3. Results

### 3.1. Population Characteristics

Strength data were missing for two participants in the RA-EMG group and 30 participants in the CONV Group. Similarly, data for ROM were missing for one participant in the RA-EMG group and one participant in the CONV Group (Figure 1). Seventy-three participants (50 males and 23 females) aged 27–72 years (47.7 ± 9.1 years) were allocated to the RA-EMG group, while 44 participants (28 males and 16 females) aged 32–61 years (45.3 ± 7.2 years) were in the CONV group (Table 1). The RA-EMG group performed 19.0 ± 4.3 training sessions in 51.0 ± 13.9 days, and the CONV group performed 20.6 ± 4.7 conventional training sessions in 55.8 ± 25.5 days. At baseline, subacromial pain syndrome was diagnosed in 81.8% and 81.9% of the CONV and RA-EMG groups, respectively. No significant difference (*p* > 0.05) was observed comparing age, training duration and number of sessions between groups (Table 1).

### 3.2. Absolute and Normalized Shoulder Flexion Strength Gain

**Figure 3:**
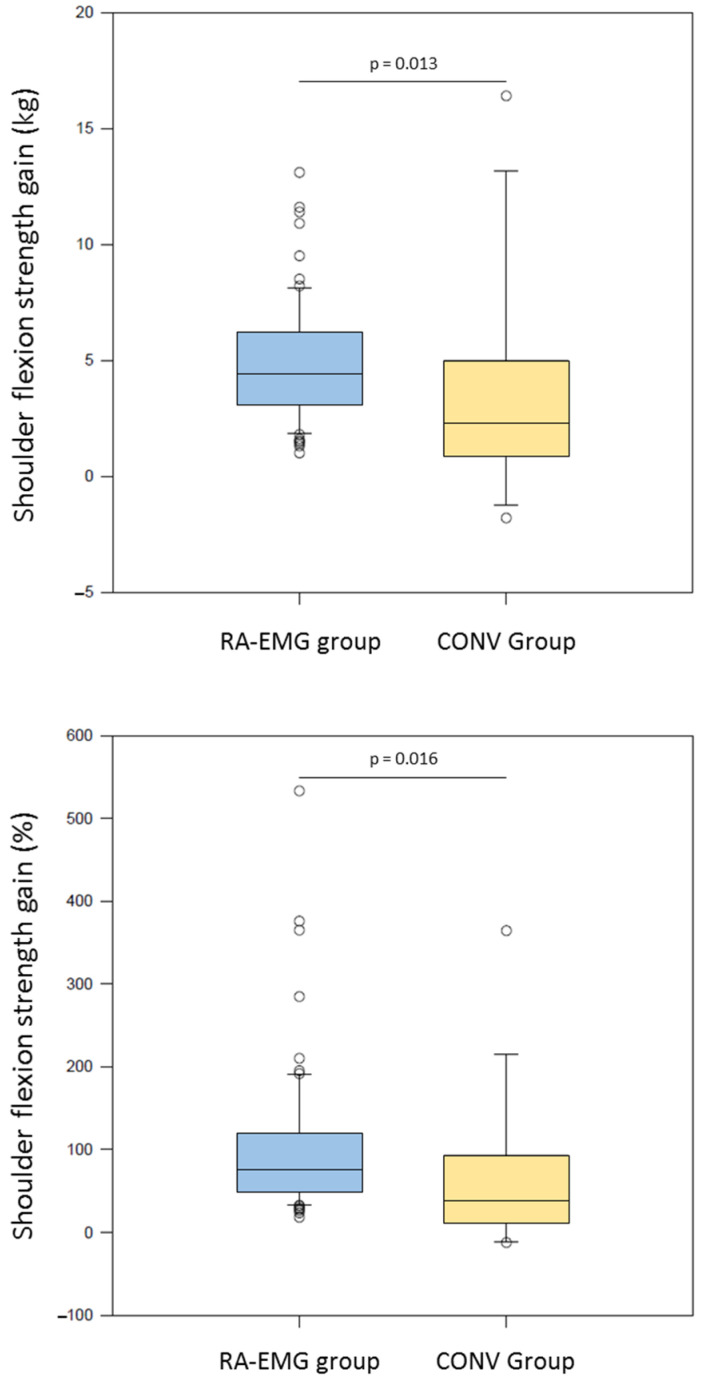
Box plots of the shoulder flexion strength gain expressed in kg (**upper panel**) and percentage (**lower panel**) for the RA-EMG group (blue) and the CONV group (yellow).

The RA-EMG group showed significantly greater absolute strength gain than the CONV group after the training program (4.45 [2.6;6.15] kg vs. 2.3 [0.90;4.775] kg, U = 761, *p* = 0.013) (Figure 3, upper panel). In addition, significantly greater benefit for normalized strength was observed in the RA-EMG group (77.5 [51.3;119.1] %) in comparison with the CONV group (39.1 [16.6;89.2] %, U = 755, *p* = 0.016) (Figure 3, lower panel).

### 3.3. Absolute and Normalized Shoulder Flexion ROM Gain

**Figure 4:**
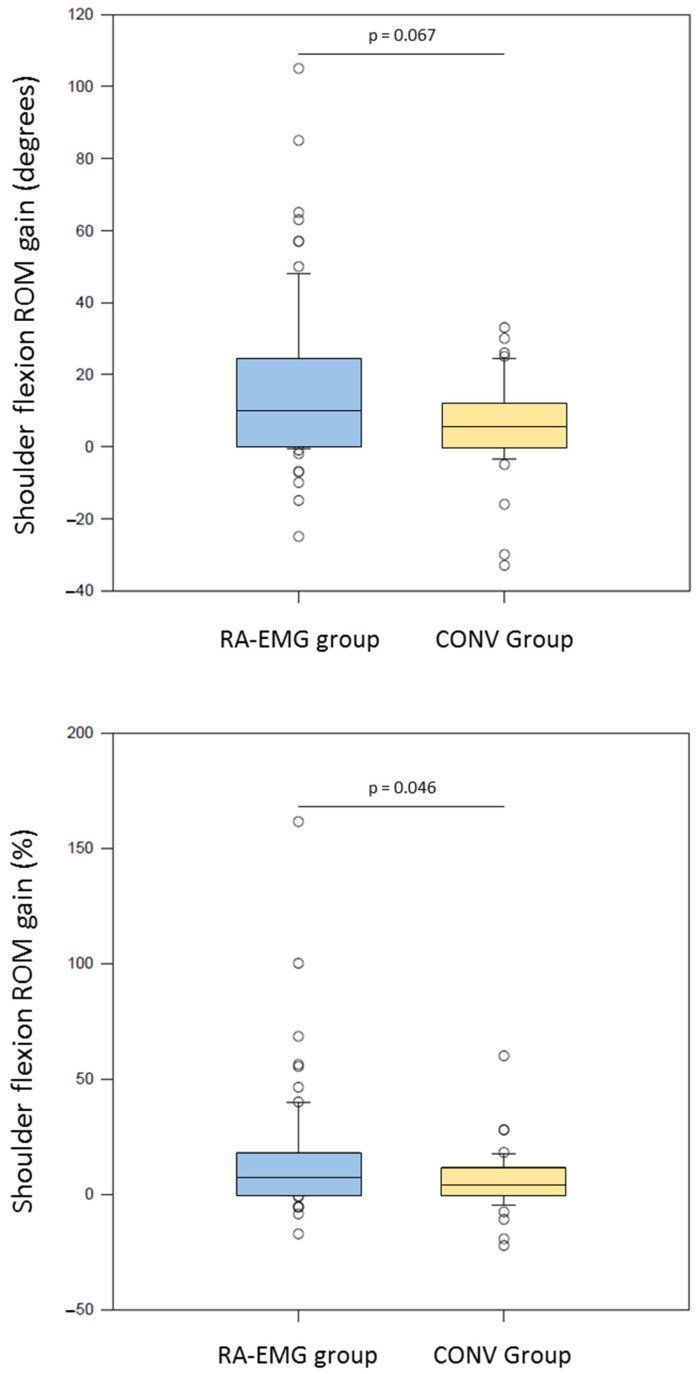
Box plots of the shoulder ROM gain expressed in degrees (**upper panel**) and percentage (**lower panel**) for the RA-EMG group (blue) and the CONV group (yellow).

After the training program, the RA-EMG group showed a trend to greater absolute gain of ROM than the CONV group (10.0 [0;24.3] degrees vs. 5.5 [0;12.0] degrees, U = 1931, *p* = 0.067) (Figure 4, upper panel). In addition, a greater benefit in normalized ROM was observed for the RA-EMG (7.4. [0;17.7] %) than the CONV group (4.6 [0;10.8], U = 1907, *p* = 0.046) (Figure 4, lower panel).

## 4. Discussion

This study showed that a robotic-assisted training program combined with EMG biofeedback led to greater gain of shoulder flexion strength and a trend in range of motion compared to a conventional strength program. This study provides promising results, showing the added value of a robotic-assisted program combined with an EMG biofeedback device in an MSD population.

By using a similar device in an 8-week strength program (three sessions/week) in healthy adults, Bui et al. [33] reported up to 26% strength gain in both right and left shoulder flexion. While relative gain was reported in healthy adults, our study showed that a robotic-assisted device combined with EMG biofeedback in an MSD population provided up to 77.5% strength gain compared to the 39.1% observed with conventional rehabilitation. In a recent systematic review, Argut et al. [32] indicated that EMG biofeedback can effectively help improve quadriceps strength. While the pain associated with MSD [4,5] may contribute to decreased corticospinal excitability [39,40], EMG biofeedback has been shown to enhance motivation, enabling patients to achieve desired levels of muscular contraction and improving treatment adherence [32,41]. Gumaa and Rehan Youssef [42] showed in their literature review that evidence of the effectiveness of virtual reality or augmented environments is promising for shoulder impingement syndrome, creating a distraction from the painful region [43] and supporting the idea that more playful and personalized rehabilitation is beneficial for the patient. In addition, telerehabilitation might be considered to improve MSD management [44], helping to increase the number of rehabilitation sessions outside the clinical environment.

Associated with strength, ROM has been identified as a critical component in MSD shoulder rehabilitation [19,45]. In a systematic review and meta-analysis, Steuri et al. [19] determined the effectiveness of conservative interventions for range of motion in 6093 adults with shoulder impingement through 113 trials. This study reported that specific exercise therapy was superior to non-specific exercise and that manual therapy plus exercise was superior to exercise only. The literature has failed to report any superiority of the EMG biofeedback approach to manage ROM compared to conventional approaches [32]. In line with these findings, our results showed that a specific guided exercise rehabilitation program provided a trend of higher ROM outcomes than conventional therapy. While the AME device was specifically dedicated to recording and displaying muscular activity, ROM would be less sensitive to EMG biofeedback. The AME device might present only a slight added value for managing ROM in patients with MSD syndrome and might be enhanced by combination with manual therapy.

In addition to potentiating strength and ROM rehabilitation, EMG biofeedback was used to ensure adequate activation of the muscle involved in a given exercise [46,47,48,49,50,51]. The AME device adjusted the level of resistance force until total passive movement [52], to provide constant EMG activity [53], allowing us to take into account fatigue components during a training session. Thus, maximal EMG activity was determined before each training session considering the current strength capacity and achievement over the training period program in compliance with the recommendations of active and progressive rehabilitation for shoulder MSDs [13,54,55]. Previous studies have argued that EMG biofeedback increases patient motivation [43,44] and facilitates patient compliance by modulating muscular activity in real time 48]. In line with this finding, the AME device and EMG activity feedback (i.e., a standardized measurement protocol) contributed to individualization, person-centered care and a participative rehabilitation approach [56,57,58,59,60,61].

### Limitations

Even though our study presented evidence favoring RA-EMG in management of shoulder MSDs, some concerns should be considered. First, only strength and ROM were clinically assessed, while it has been recommended to evaluate pain and functional disability [19]. Similarly, abduction and external rotation should be treated to restore and involve agonist and antagonist muscles. In addition, strength measurement was performed using two different systems: the AME dynamometer for the RA-EMG group and a conventional dynamometer for the CONV group. Both devices employ similar technology, relying on a Wheatstone bridge to assess strength. The reliability of strength measurement with the AME device has been established in patents (US patents [US.8.262.541] and [US.8.187.152]; Canadian patent [CA 2714914]). While we cannot entirely exclude slight differences between device measurements, it is reasonable to assume that these differences did not significantly impact our results. While the final goal is to return to work [62], the rehabilitation period before returning to work could also be considered a key endpoint. While our study included various MSD pathologies, direct comparisons between different populations were not conducted due to the overrepresentation of subacromial impingement syndrome (81.8%). Nevertheless, our findings suggest that a robotic-assisted training program combined with EMG biofeedback may be beneficial for individuals with diverse MSD pathologies. Thus, care should be taken when generalizing the current results to patients not having subacromial impingement syndrome. Finally, the cost and size of the device may limit its accessibility and adoption in rehabilitation centers. Future prospective randomized controlled trials should be conducted to strengthen the level of evidence, particularly by comparing the efficacy of rehabilitation interventions across various MSD pathologies.

## 5. Conclusions

The current retrospective cohort study showed that a robotic-assisted device combined with EMG biofeedback provided greater strength and a trend of improvement of range of motion shoulder flexion after an 8-week rehabilitation period compared to a conventional approach in patients with shoulder MSDs. EMG activity as biofeedback offered a tailored rehabilitation program with constant effort by automatically adjusting the level of resistance based on a specific EMG target and may have enhanced patient motivation. Future research with a randomized controlled design should determine the potential added value of a robotic-assisted device combined with EMG biofeedback on abduction and external rotation.

## Figures and Tables

**Table 1:** Baseline characteristics for the RA-EMG and CONV groups.

	RA-EMG Group N = 73	CONV Group N = 44	*p* Value [t Value]
Sex, N (%)			
Men	50 (68.5)	28 (63.6)	0.940
Women	23 (31.5)	16 (36.4)	0.169
Age in years (mean ± SD)	47.7 ± 9.1	45.3 ± 7.2	0.092 [1.700]
Training duration in days (mean ± SD)	51.0 ± 13.9	55.8 ± 25.5	0.188 [1.324]
Number of sessions in days (mean ± SD)	19.0 ± 4.3	20.6 ± 4.7	0.064 [1.872]
Classification of diseases, n (%)			
Subacromial pain syndrome	59 (81.9)	36 (81.8)	-
Shoulder dislocation	4 (5.6)	5 (11.3)	-
Other shoulder MSDs	9 (12.5)	3 (6.8)	-
Flexion shoulder strength, kg (mean ± SD)	6.2 ± 3.5	7.6 ± 3.9	0.41
Range of motion, degrees (mean ± SD)	141.8 ± 31.2	138.3 ± 34.6	0.62

MSDs: musculoskeletal disorders; SD: Standard Deviation.

## Data Availability

Data are contained within the article.
